# The Story of the Insane from Year to Year

**Published:** 1895-03-30

**Authors:** 


### THE STORY OF THE INSANE FROM YEAR TO YEAR.

(7th Series.)

### EXETER BOROUGH ASYLUM.

On January 1st there were 340 patients in this asylum, and on December 31st the number had risen to 345. There were 81 admissions, 28 recoveries, and 25 deaths. The recoveries work out at 46·6 per cent. on the admissions, and the deaths at 7·3 on the average number resident. Domestic trouble caused the insanity in 6, drink in 11, and hereditary influence in 19. Cerebral and spinal diseases caused death in 7 cases, phthisis in 4, and diarrhœa in 1. When referring to the resignation of Mr. Commissioner Cleaton, Dr. Rutherford says: "His long and varied experience of asylums and the insane rendered his criticisms fair and marked by common sense, and his reports of asylums were never marred by ill-considered and petty fault-finding." The weekly cost of maintenance was 10s. 8d. per head.

---

### CUMBERLAND AND WESTMORELAND ASYLUM, CARLISLE.

A decrease of one patient occurred during the year 1893, there being 580 on the 1st of January and 579 on the 31st of December. There were 178 admissions, 81 recoveries, and 48 deaths. The recoveries stand at 45·5 per cent. of the admissions, and the deaths at 8·1 of the average number resident. Hereditary tendency accounts for 65 of the year's admissions, previous attacks for 46, drink for 22, domestic trouble for 11, and religion for 10. Cerebral and spinal diseases were the causes of death in 13 and consumption in 4. As already stated, the percentage of recoveries was 45·5, and Dr. Campbell points out that if transfers be excluded in the calculation the rate would rise to 49·3, and on looking over Table III. we find that this high recovery rate is by no means exceptional. During the first six years of the existence of the asylum the recoveries were not high, but lately they have often been as high as 50 per cent. and even above it. This may be creditable to the medical staff, but it will have the inevitable result of increasing the insanity in Cumberland and Westmoreland during the next generation. Dr. Campbell is fortunately able to give a favourable report of the conduct of the staff, and he adds, "in a large establishment like this there are always some officials who incline to take life, its cares and responsibilities, more lightly than others do." One hardly knows whether to envy or blame these easy-going philosophers, but it is certain that asylum work is apt to be a little irksome at times, and members of the staff may be pardoned for "knocking off" a little now and then. The Committee say they are perfectly satisfied with the condition, management, and superintendence of the asylum. The weekly cost of maintenance is 9s. per head.

